# The significance of communities of practice: Norwegian nursing students' experience of clinical placement in Bangladesh

**DOI:** 10.1002/nop2.15

**Published:** 2015-04-06

**Authors:** Wanja Jørgensen, Hans Hadders

**Affiliations:** ^1^ Faculty of NursingSør‐Trøndelag University CollegeTrondheim7004Norway

**Keywords:** Clinical placement, clinical supervision, communities of practice, legitimate peripheral participation, nurse education

## Abstract

**Aim:**

The purpose of this study was to gain understanding of Norwegian students' experience of learning in clinical placement in Bangladesh without formal one‐to‐one supervision, by a personal mentor in the ward.

**Design:**

Using focus group interviews with bachelor nursing students we explored the significance of ‘communities of practice’ in nursing practicum abroad, socialization and knowledge transfer.

**Method:**

Seven third year bachelor nursing students enrolled in a clinical placement programme in Bangladesh participated in focus group interviews prior to their departure to Bangladesh, during their stay in Bangladesh and after their return to Norway.

**Results:**

The Students’ marginality and ‘peripheral participation’ triggered insight and reflection. The challenging but advantageous position of the peripheral students was heightened further due to the lack of one‐to‐one supervision in the clinic. Their previous experience with problem based learning and group learning was an asset that made them more resilient and helped them to cope.

## Introduction

Clinical placement is an important arena for undergraduate nursing education. Access to the clinic gives students an opportunity to acquire practical skills and contextualized knowledge in bed‐side situations. In the European context, nursing training and clinical placement traditionally took the form of an apprenticeship model where students spent most of their time in a supernumerary capacity working alongside qualified nurses in hospital wards. Such knowledge transfers were typically hierarchical, with little personal supervision and often based on unreflective copying of role model's task performance (Spouse 1998a, 2001, Scott 2013).

Presently, in most cases, Norwegian nursing students are allotted a personal clinical mentor, a ward nurse, responsible for supervision and guidance during clinical placement. Mentors are important guides and gatekeepers to their professional communities (Spouse 2003, Scott 2013). In Norway, the clinical mentor has regular scheduled meetings together with the student and a tutor from the Nursing Faculty. The clinical mentor and the tutor are responsible for regular and final assessment of the student's performance. This paper presents an oblique view on clinical placement in the Norwegian context and reports how Norwegian nursing students' learn, socialize and cope in clinical placement in Bangladesh without the formal one‐to‐one clinical supervision by a personal clinical mentor in the ward that they are used to in Norway. (Clinical Placement in Bangladesh is akin to the traditional apprenticeship model in Norway with little personal supervision in the ward). First, a brief background to nurse education in Norway is given. Second, the analytical approach to communities of practices is explained. Third, the design of the study is outlined followed by findings, interpretation and a discussion. The article ends with some concluding remarks.

### Background

Clinical practicum has been part of nursing education since establishment of institutional nursing training in Norway. Many nursing schools where owned and financed by municipal hospitals and there was a strong link between the two institutions. In the 1960s, this set up was abolished and the management and financing of nursing education was taken over by the central government (Jensen 2006, p. 5). During the 1960s, 75% of the nursing training still constituted of clinical placement. Presently, approximately 50% of the nursing training constitutes of clinical placement (National Curriculum for Nursing Education 2008).

The current Norwegian nursing educational paradigm has a strong focus on academic, analytical, assertive and reflective qualities (National Curriculum for Nursing Education 2008). A common strategy to support reflective qualities and bridge the potential gap between theory and practice in nursing education has been the application of problem‐based learning (PBL). Student active learning methods, self‐directed learning and PBL are regular features of the Bachelor nursing programme at the Nursing Faculty, Trondheim University College Faculty (National Curriculum for Nursing Education 2008).

#### Norwegian nursing students clinical placement in Bangladesh

In the wake of globalization and a global health focus, international clinical experience in low and middle‐income countries has become a popular strategy to enhance cultural reflexivity, awareness and competence among nursing students in high‐income countries. ‘Cultural awareness’ is widely recognized as an essential component for nursing students in contemporary multicultural societies (Tabi & Mukherjee 2003, Maltby & Abrams 2009, Reid‐Searl *et al*. 2011). However, several authors challenge the claim that international placement, or so called ‘immersion experiences’ in low and middle‐income countries, immediately transforms participants into cultural experts (Culley 2006, Harrowing *et al*. 2012). Furthermore, at worst, ‘immersion experiences’ can become voyeuristic health tourism. Nevertheless, there is an agreement that international placements can provide an important awaking that can deepen further awareness about socio‐cultural differences. This awareness can be fostered and enhanced through various supportive educational interventions during the entire graduate educational process. Several authors underscore that education about ethnicity, racism, socio‐political perspectives and social determinants of health are crucial for students to make sense of their ‘immersion experiences’ and benefit from these experiences in their future professional practice (Culley 2006, Harrowing *et al*. 2012, Mkandawire‐Valhmu & Doering 2012, Racine & Perron 2012).

Since 2006, the Nursing Faculty at Sør‐Trøndelag University College has sent a small number of third year Bachelor programme (BA) students to International centre for diarrhoeal disease research Bangladesh (ICDDR,B). Khan, Pietroni and Cravioto underscore that over half a century ‘ICDDR,B … has played a significant role in research and innovation in the global public‐health arena, historically in the areas of child health and diarrhoeal diseases and more recently in broader care, including maternal and child health and HIV/AIDS’ (Khan *et al*. 2010; 533). Students have enrolled in the International Field Experience programme at ICDDR,B. Initially, the third year BA students spent a month in various wards collecting information for their bachelor thesis. During autumn of 2011, a pilot project was launched and seven bachelor students completed 7 weeks of clinical placement in hospital wards at ICDDR,B in Dhaka and Matlab, Chandpur district.

Prior to departure, the seven students participated in an obligatory course in Global Health consisting of 4,5 international credit points (ECTS) and they also received an elective course organized by supervisors at the nursing college. The latter course was meant to give the group some knowledge and tools in preparation for their placement. Among other things, students were briefed through lectures and articles about; disease panorama, history of Bangladesh and ICDDR,B, various challenges, security and nursing role in Bangladesh and the traditionally low status of nurses in Bangladesh (Zaman 2004, 2009, 2013, Hadley & Roques 2007, Hadley *et al*. 2007).

#### Communities of practice: analytical approach

Using the term ‘communities of practice’ coined by Wenger (1998), we explore the significance of communities in nursing practicum abroad, socialization and knowledge transfer. Initially Wenger coauthored an important work together with American social anthropologist Jean Lave, with the title *Situated learning, legitimate peripheral participation* (1991). This work chalked out their challenge to what they perceived as a traditional decontextualized classroom approach to learning and invoked a sociological analysis of learning *in situ*.

### Situated learning

Lave's theoretical perspective of learning initially developed from her ethnographic research on Vai and Gola tailors’ craft apprenticeship in Liberia, West Africa. Through her research focus on apprenticeship, Lave discovered the complex and inherently social nature of tailor‐craft knowledge transfer. She found that apprentices learnt many complex lessons at once and that they were not merely, ‘learning by doing’, or reproducing the mechanics of tailoring craft. In addition to mastering the technics of the craft, the apprentices ‘were learning relations among the major social identifies and divisions in Liberian society which they were in the business of dressing’ (Lave 1996, p. 151).

The genesis of Lave and Wenger's concept ‘peripheral participation’ started with an investigation of situated learning via their focus on apprenticeship. To explore the inherent complexities of work place learning and socialization, Lave and Wenger developed an analytical tool with which to illuminate knowledge transfer and the many nuances and varieties of master‐apprentice relations in communities of practice. The manifold trajectory from being a newcomer to becoming a full‐fledged member of a community was explored with their analytical concept ‘legitimate peripheral participation’ (Lave & Wenger 1991).

### Legitimate peripheral participation

The concept ‘legitimate peripheral participation’ (LPP) is intended to be taken and applied as a whole to enable a wider social view of apprenticeship and knowledge transfer. What needs to be carefully scrutinized and analysed is the gradual access to a community, what kind of participation is legitimate, to what degree and how participation and non‐participation changes over time as the newcomer gradually becomes incorporated in the community as a member.

The theoretical approach supplied by Lave and Wenger is especially apt for an analysis of nursing education, with its foundation in professional ‘guild like’ learning institutions, with a strong emphasis on bedside apprenticeship ‘*in situ*’. Their concept ‘LPP’ is especially effective in an analysis of professional apprenticeship, with formalized face‐to‐face learning trajectories, which span over longer periods, as is the case in 3‐year Bachelor nursing programmes. Nursing, as a profession, with extensive amount of obligatory time spent in clinical placement, represents solid professional communities of practice with considerable long‐standing tradition (Spouse 1998a,b, Ranse & Grealish 2007, Thrysoe *et al*. 2010, White 2010, Scott 2013).

Lave and Wenger's model presents a novel sociological view on knowledge transfer via obligatory apprenticeship that emphasize interactions and learning, not only between master and novice, but between other group members, such as peers and team members. Thus, the concept LPP provides an analytical framework for bringing together theories of situated practice and theories about production and reproduction of social identity and social order (Lave & Wenger 1991). In a wider sense, Lave and Wenger explored the social reproduction of communities of practice (1998).

### Communities of practice

Communities of practice (CoP) are more or less formal arenas for negotiating learning, meaning and identity. Initially Wenger gives a simple micro definition of the basic concept. CoPs are formed by groups of people who share a concern for something they do and learn how to perform this practice better as they interact regularly (Wenger 1998). For Wenger, learning in situated practice can be traced to three dimensions, which constitute these communities. First, members interact and establish relationships and norms for interaction through ‘mutual engagement’. Second, members are tied together by an understanding of a sense of ‘joint enterprize’. Finally, members establish a shared ‘repertoire of resources’ and jargons over time, such as routines, language, tools, stories and a common sense of humour (Wenger 1998, 73–85). Wenger's blanket definition covers a wide perspective of CoPs, ranging from small informal and short‐lived Cops to permanent professional CoPs.

There are a smaller number of published studies, which make use of the LPP and CoP concepts in research about clinical placement in nursing education. Nevertheless, it is somewhat surprising that Lave and Wenger's concepts have not been more commonly applied in this area (Spouse 1998a,b, Ranse & Grealish 2007, Grealish & Ranse 2009, Thrysoe *et al*. 2010, 2012, White 2010). However, there are a few applications of CoP used to analyse innovation, knowledge management and collaboration between nursing institutions and academia (O'Connor 2006, Andrew & Ferguson 2008, Andrew *et al*. 2008).

In this article, for the sake of clarity and precision, we anchor our application of CoP in the original definition of the concept as outlined above (Lave & Wenger 1991, Wenger 1998). In our analysis of Norwegian nursing students' clinical placement in Bangladesh we mainly focus on knowledge transfer, group apprenticeship, learning facilitation, sponsorship, students' role as legitimate learners, their socialization and how they handle challenges as they aspire to join the CoP they encounter during their learning trajectories.

## The study

### Aim

The purpose of this study was to gain understanding of students' experience of learning in clinical placement in Bangladesh and to explore what resources and personnel they make use of during their learning process.

### Data collections and methods

This study was exploratory, using qualitative data. Seven third year BA students enrolled in a clinical placement programme in Bangladesh were invited to participate in focus group interviews (Barbour 2010, Halkier 2010). Focus group interviews with the seven students in one single group were conducted prior to their departure to Bangladesh, during their stay in Bangladesh and after their return to Norway. All students agreed to participate. Interviews were approximately 90 minutes in length. The first author moderated the focus groups and second author assisted and audio recorded the sessions. The interviews were facilitated by open‐ended questions with low moderation and all participants had an opportunity to vent their anticipations, views and experiences of learning in practice. Participants were asked to describe their expectations, experiences of socio‐cultural encounter and how they learnt and coped with challenges and the impact of their clinical placement (Halkier 2010). Other sources of data included written individual learning objectives for clinical placement, written individual assignments and reflections about their experience and achievement of learning objectives.

### Ethical considerations

Approval for the study was obtained from the institutional research comity at the Nursing Faculty. An ethical issue was the potentially biased relationship between the researcher/teacher and students. This issue was managed by presenting written and oral information about the study to students before recruitment. Participation was voluntary and withdrawal from the study at any time had no consequence for the students. Written informed consent was sought for all interviews. In addition, all students were provided with written information about the study and the conditions for their involvement. Participants were assured that no individual names would be used in any reports of the study. The transcripts were identified by code number. Student assignments and written individual learning objectives were rendered anonymous.

### Data analysis

The interviews were transcribed by an assistant. Transcriptions as well as audio version of interviews have been used during analysis of the interviews (Strauss & Corbin 1990). The datasets were read and reared several times independently by authors. The authors coded the data by hand and content analysis was used to identify major themes and patterns (Kvale 2009). The written individual learning objectives and written individual assignments were read and reared several times independently by authors and served as complementary data in the analysis of the interviews. Excerpts appearing in this article have been translated into English by second author.

## Findings

Themes were identified in three different phases of the placement and the learning process. Major themes were expectation, anticipation and apprehension, encountering and cooping, learning outcomes and challenges coming home. Each theme is illuminated by quotes from the participants and elaborated.

### Expectation, anticipation and apprehension

The first interview was conducted a few days before students' travel to Bangladesh. Prior to departure, the students expressed their anticipations about how they expected to be received by the Bangladeshi nurses and how they expected to achieve their learning objectives during their clinical placement. Initially they mentioned apprehensions about, on the one hand, being excluded from performing ‘hands on’ clinical practice and on the other hand, being expected to perform ‘hands on’ clinical practice without relevant preparation and guidance. They asked themselves if their clinical placement would mainly consist of observation or if they would get the opportunity to participate and contribute in the clinic.

Students discussed the significance of predictability about their student role. They realized that many aspects of their role would remain uncertain before their arrival in Bangladesh. Nevertheless, they had clear expectations about their student role. The following aspects emerged and exemplified what they anticipated; to be responsible for their learning process, be more independent, be brave, seek out learning situations, be flexible and feel confident academically and personally. They also acknowledged the importance of group fellowship as a key for their success as individual students. Group affinity was acknowledged as a significant anchorage for their learning. Students emphasized three prerequisites for their group to function; showing mutual respect, open communication and fellowship. Respect for diversity and differences among the group members were considered important to achieve common as well as individual learning goals.

The students mentioned various strategies they thought could help them in their progress along their learning trajectories. They also mentioned concerns about expected challenges during their learning process; at both personal and professional level and how they expected to meet these challenges. On the one hand, they expected lack of formal one‐to‐one supervision in the ward to become a challenge. On the other hand, they did not perceived lack of personal supervision exclusivity negative.

One student underscored that: ‘I expect to participate even though I know that we will have to do a lot of observation…We have to grasp all opportunities we get and we have to be proactive and find situations where we can latch on to a nurse who can teach us something…’

Another student argued that they all would soon become graduated nurses and would have to practice nursing independently.

Students expected the group to strengthen their ability to cope with the overwhelming clinical encounters, in absence of a personal supervisor. They all emphasized the importance of establishing a fellowship and one student stated: ‘belonging to a group will be a safe base and will give us feeling of fellowship in an alien environment … and possibility to cope with expected challenges’. All students agreed with the following statement made by another student: ‘… It will be ever so important to use each other, especially when it comes to reflection and to teach each other on the basis of our own experience and support each other in seeking out learning situations’.

One student mentioned the importance of feeling secure: ‘We have spent considerable time to establish the group as a safe base…’ Another student added: ‘… then one feels secure and can tackle things together. I think this is a super foundation for a successful clinical placement’.

In addition, they expressed a desire to make a difference in the clinic and to contribute with their knowledge and effort which they hoped could be useful for the Bangladeshi nurses. On the one hand, one student underscored the students’ responsibility for their own learning: ‘We must take the initiative find appropriate learning situations and are responsible for our own success’. On the other hand, the students had expectations about the nurses and facilitators willingness to show interest and take initiatives to contribute to students’ learning.

One student voiced her apprehension about various challenges they would meet in the clinic: ‘… we will certainly encounter an awful amount of challenges … just imagine; we have become so good at recognizing ethical dilemmas and we are fully loaded [with such preconceptions] from our training in Norway …’ All students agreed when one student underscored that: ‘We must be allowed to be critical and we shall not, kind of, accept everything because we are abroad …’

Students considered the Bangladeshi facilitators’ role important to help them achieve their learning objectives. They realized that mentoring would differ from what they were used to in Norway. Nevertheless, students expected to be included in the team of local nurses. One student underscored: ‘I hope we are expected and will be well received as a student and that someone will take care of me.’ They also mentioned building good relations with the staff at the hospital, especially with the nurses: ‘I want to be culturally sensitive and establish good relations with the staff at the hospital…and get a feeling of affiliation [with them]…’

They also mentioned the supportive role of their Norwegian Nursing College as an important prerequisite for learning. They expected their Nursing College to be a ‘communication‐central’ to provide administrative support and safeguard the academic framework adequately in planning and for further follow‐up. Communication between home institution and host was expected to be a continuous and an ‘open line’ of communication in case of any special or urgent needs.

### Encountering and coping

The second interview was conducted in Bangladesh when the students were about midway through their clinical placement. They had spent the first 2 weeks in clinical placement at the ICDDR,B hospital in Dhaka and 1 week in a local ICDDR,B hospital at community level in Matlab, Chandpur district south of Dhaka. We interviewed the students when they had returned to Dhaka and resumed their placement there.

Themes that emerged were encountering and coping during students' learning process. Students commented on experienced challenges, cultural encounters and how they manage initial overwhelming impressions overall in the clinical setting. They acknowledged the significance and the strength of being a group. The students considered ample access to the clinical setting essential for their understanding of the complexity in the hospital. The group members experienced the initial encounters differently.

One student described the first meeting with Dhaka as chaotic: ‘…when I arrived at the guest house I got panicky and felt trapped in the room’. However, another student described her first meeting as predictable: ‘I had experienced situations like this before in similar countries and personally I think it was a big advantage’.

The first week in clinical placement at ICDDR,B Dhaka hospital was more or less difficult to manage for all students. They commented that initially, without a personal mentor in the ward, their new student role required them to be constantly ‘switched on’ and on alert, responsible for their personal learning. This was perceived as exhausting. As one student expressed: ‘I felt like standing outside reality and all passed as on the cinema without options to leave…I had no control…’ Another student supported this statement and added that some local nurses did not take initiative to include them in the clinic and the students had to be ‘super engaged’ and make a major effort daily to be considered or being seen at all.

However, the overall group facilitation, organization and support they received from ICDDR,B were high above their expectations: ‘I had expected that we would be more left to our own; practically…[On the contrary] we have received incredible support and coaching’. Students expressed that belonging to a group had a great significance for them and how they coped emotionally, facing challenging encounters during their first weeks in clinical placement: ‘Even if I had read that it would be different with lots of sounds and people everywhere, it is something else when you experience it physically with your own body. Even when you take a break at the Hospital it is still a very intense as you are there [at the hospital premises]’.

Students commented on their mental state during the first week of clinical placement: ‘… maybe we were a bit behind during the first week … we had to use a lot of time to reflect upon what we were facing…we returned [to the Hotel] at five, had some food and discussed the day's experiences until nine in the evening when we tried to get some sleep until we went to work again next day…’ Another student commented: ‘I can't remember that we laughed during the first week; we were very serious all the time’.

One student expressed her frustration:

I think it has been very difficult to spend time in the hospital, as I have no experience like this before. I have often been frustrated and sad because it is difficult to know what to do about things and how to influence and what it is that we just have to accept…One feels powerless and wants to be culturally sensitive and establish good relations with those around you, at the same as it is burning inside of me to voice my opinion about things…

Another student expressed the indispensable group support with these words: ‘If it had not been for the support from those around me [in the group] I would have gone home…’

Gradually the students realized the need for a break as a necessity to get control of the situation and to obtain more predictability in their daily life. As one student mentioned: ‘We decided to have some fun and now we can manage to go out to have an ice‐cream and have funny conversation and we can laugh…’ The group confirmed: ‘to be able to learn you need to have fun as well.’

A significant turning point for students’ learning process took place when they changed their placement to the smaller community hospital in Matlab where the students lived in a guesthouse at the hospital premises. In opposition to the unfamiliar diverse situation at the large ICDDR,B hospital in bustling Dhaka, they easily got an overview of the small familiar rural hospital, the patients and staff at Matlab. One student expressed: ‘The placement in Matlab was a turning point for me and most important; we were expected, received and warmly greeted [when we arrived] and we were immediately considered a part of the team’. Another student commented:

During Matlab hospital placement everything took another turn for me; this was a small hospital and much easier to get contact with people and dare to let oneself lose. I ventured to communicate with patients and nurses and this made it so much easier to return to Dhaka hospital…

The sponsorship in the Matlab wards allowed them to get sufficient clinical experience and enabled them to feel safe enough to take initiatives and perform procedures on their own. Another significant prerequisite mentioned was that the clinical facilitators in Matlab wards seemed to be prepared for their arrival. One student elaborated: ‘I perceived that the staff was well informed and prepared to receive us; they were well informed about our placement objectives [expectations] and our student role was settled in a way…’. Another student added:

At Matlab there were 6–7 nurses that we spent time with most of the time. We came to like them very much. They phoned us at night whenever there was a delivery [at the ward]. These were much more reciprocal relationships. Sometimes I feel that the relationships at Dhaka [hospital] mostly were one‐way relationships.

Another student commented that at Dhaka hospital the pace of work was much more hectic and some nurses sometimes were reluctant to include students whereas others were good at including the them in the clinic: ‘More or less they take time to answer if we ask them [for advice] even if they are quite busy…I have got the impression that not all nurses are informed about what we do here and why we are here.’

All students expressed that that the communication at Dhaka hospital was quite difficult and the language barrier, especially with patients, was overwhelming in the beginning. They expected to contribute in the clinic, but the language barrier was a big obstacle. As one student expressed: ‘….it's quite hard when someone try to talk with me and I couldn't answer and I felt so useless…’ Because of the obstacles relating to language communication, they became more aware of their non‐verbal communication. Improving their non‐verbal communication became an important a learning objective for several students.

Another student added: ‘personally, I have learnt to be more conscious about my own expressions or body language… and to become more competent in communication was one of my learning objectives’. Several students also mentioned the revelation that their experience of standing on the outside, or being excluded in a sense, could be transferred to the experience of minority and immigrant patients’ experiences in Norwegian health institutions.

When asked by the interviewer in what way they had learnt one student: ‘The first thing that strikes me, if I shall point at something that is different than in Norway in a positive way, is the way I have learnt here; that is that I have learnt together with someone else (e.g. a fellow student instead of with a personal supervisor)’. This student elaborated further:

We have been very good at reflecting outside the clinic. I think that is a good way of learning…to have someone next to you when you ask a question about something you cannot understand…a fellow student with which to double check with your understanding and to share an experience…

### Learning outcomes and challenges coming home

The third interview was conducted in Norway approximately 2 weeks after the completion of the students' clinical placement in Bangladesh. The main purpose of the interview was to get a deeper insight into their learning process over time. Besides exploring the students learning outcomes, if and how they had achieved their learning objectives, students were asked to considerer their clinical placement in hindsight from a meta‐perspective. Students commented extensively about their learning objectives and how they managed the challenges, they had met in the clinic. During this final interview, instead of focusing on the learning process, students talked more about achieving their learning objectives related to outcomes, nursing skills and nursing identity in a broader perspective.

As in the second focus group interview, they described their learning trajectory, from being ‘peripheral legitimate non participants’ to becoming ‘legitimate peripheral participants’. They also described instances of dissent and disagreement with local nursing practice. They felt that their student role had been strengthened after placement at Matlab hospital. This encouraged them to be more participative, take more initiative and to be more independent in Dhaka hospital. They commented challenges and success in reaching their learning objectives, both related to individual objectives, as well as, the learning objectives presented in their curriculum. In addition, the students mentioned the importance of self‐directed learning and gaining knowledge through extensive self‐study, reflective log writing and access to Internet and the ICDDR,B research library during their placement.

Some examples of the learning objectives were; caring in new cultural setting, knowledge and skills related to cultural sensitivity, learning about health in a global perspective, learning about new disease panorama related to diarrhoeal and infectious disease, skills in practical procedures, administering intra venous fluid, become more skilled in nonverbal communication. They highlighted their personal achievements obtained during their clinical placement as becoming; more independent, more active, more reflective, more courageous and proactive in learning situations. They all agreed that their new knowledge could be transmitted to the Norwegian context in their future role as registered nurses. The students mentioned the communication and language barrier as challenging as they strived to obtain interaction with staff, patients and relatives. The lack of means of communication was considered a major obstacle for their inclusion in the local nursing community.

The reversed culture ‘shock’ the students experienced as they returned to Norway was difficult to cope with and this resulted in a break in their learning trajectory. The fact that their group was dissolved as they returned and all students were busy with other pressing educational tasks, which prevented time and space for further group debriefing was experienced as difficult. The students mentioned that sufficient time and an appropriate arena, allocated by their nursing college, could have avoided their feeling of interruption. Students also expressed difficulty to find reference persons, which could replace their group. One student expressed her feeling of not being part of the group: ‘The initial weeks [after returning to Norway] passed very quickly and I hardly met the other group members. It was a heavy experience and after 2 weeks I had a serious breakdown; I lost all my energy, I had suppressed everything …’

Another student expressed her feeling as a kind of ‘nightmare’ without the possibility to work out or debrief the homecoming encounter. One student comments difficulties in finding appropriate persons and forums to relate and discuss their experiences with: ‘people ask [politely], but they cannot understand or aren't interested enough …’ One student comment on being prepared to meet the reversed culture ‘shock’:

It was a rapid transition, but I was prepared and understood that homecoming would be more difficult, but nevertheless I had ignored this [experience] in a way. There were so many other new issues that demanded and caught my attention. I consciously decided before we travelled [to Bangladesh] that coming home to Norwegian everyday life would be a pleasure.

Despite the challenges, overall students evaluated their learning outcomes as satisfactory, professionally as well as personally. They considered their obtained knowledge relevant and transferable to the Norwegian nursing context.

#### Interpretation and discussion

As pointed out above, CoPs evolve when members of a group interact and establish durable relationships and norms for interaction through ‘mutual engagement ‘are tied together by an understanding of a sense of ‘joint enterprize’ and establish a ‘shared repertoire of resources’ and jargons over time (Wenger 1998, 73–85). Nursing communities have been organized as professional guilds and represents solid professional social groups with long‐standing lifespan. In our analysis of the significance of CoPs during students’ experiences of clinical placement in Bangladesh, we found three different major manifestations of CoPs relevant to the students learning and socialization; first their Norwegian professional nursing community, second the Bangladeshi professional nursing community and finally the proto‐CoP consisting of the seven students. As have been illustrated above, the proto‐CoP functioned as an important support for the students throughout their learning trajectories. How did the proto‐CoP originate?

Initially, the students were affiliated in a lose way as they enrolled in the nursing programme at the faculty of nursing at the University College as part of a batch consisting of approximately 200 students (part of the Norwegian nursing CoP). Prior to their application to travel to Bangladesh, in the international placement programme at the nursing college in Sør‐Trøndelag, most of the seven students did not have a close relationship as peers. As their applications were granted staff in charge of international placement facilitated the students’ group foundation actively through various elective preparatory and educational group activities. However, the students also started to meet and bond regularly on their own accord as they prepared themselves for their placement abroad. At this point, the students shared what Wenger call a specific ‘domain’, which constitutes an area of interest, a platform for their proto‐CoP (Wenger 1998). The formation of their proto‐CoP was further strengthened as they travelled together to Bangladesh and co‐resided in a guesthouse in Dhaka. On a general level, students were strongly bonded through the challenges they faced in an ‘alien’ environment as they commuted to the hospital, ate, worked together and spent extensive time reflecting and discussing their clinical experiences. The students developed a jargon, routines, language, tools, stories and a common sense of humour to coop with their experiences.

More specifically, they used techniques they had learnt doing PBL during their nursing training in Norway. They actively took responsibility for their learning as self‐directed learners. They systematized their group learning using individual log keeping, group discussion, reflection and extensive self‐study and enquiry. Through log keeping and group reflection, group members de‐briefed, consolidated and calibrated their individual experiences and fortified their fellowship and made sense of their experiences. On their own accord, they establish a so‐called PBL ‘group contract’ with rules for interaction and hold weekly house meetings at their guesthouse to contain any difference and to solve problems in the group. PBL is an acknowledged method applied to facilitate self‐directed learning, reflection and group problem solving in clinical placement and have proven to enhance nursing students' resilience (Ehrenberg & Häggblom 2007, Chen 2011).

During their placement with ICDDR,B the students were facilitated as a group. However, in the clinic they often organize themselves in pairs to support each other and learn together. At other times the students spent time in various wards alone. Nevertheless, it was as a proto‐CoP they learnt as fellow peers when they gradually accessed the Bangladeshi CoPs.

During clinical placement, a certain amount of non‐participation and marginality is normal. However, as the Norwegian nursing students entered the clinic during their first weeks of clinical placement in Bangladesh their situation were extremely marginal in several respects. First, they had to face the language barrier and the unfamiliar clinical territory; pathological content and organization. Further, the students had limited understanding of Bangladeshi cultural codes, hierarchies, student role and the local understanding of the nursing role (Zaman 2004, 2009, 2013, Hadley & Roques 2007, Hadley *et al*. 2007). Even if the students were briefed about these matters prior to their travel.

Work place learning includes many important activities and actors, which sponsor, facilitate and mediate novice learning and participation in CoP. In her work, *Learning to nurse through legitimate peripheral participation,* Spouse apply Lave and Wenger's approach and underscores the importance of mentorship and effective sponsorship for students to gain access to a CoP during clinical placement (Spouse 1998a). How were the students sponsored in Bangladesh?

On arrival in Bangladesh, students were introduced to some important gatekeepers, members of staff and clinical leaders, at a daily morning meeting at Dhaka hospital. They also received a week of preparatory programme by facilitators at ICDDR,B. The organization facilitated the students with group supervision (1:7 students) and regular debriefing outside the ward by a clinical educational nurse consultant several times a week. The students also received extensive logistical support from ICDDR,B student welfare office and from a local clinical practice leader. Weekly Skype meetings with their Norwegian teachers also facilitated students. In this respect, the students had the required sponsorship needed to grant them entry to the local nursing CoP. However, as they entered the clinic they were left to their own device.

During their first days in the clinic, the students cautiously observed activities and did not involve themselves in hands on nursing. At this point, they were ‘legitimate non‐participants’. To begin with, the so‐called ‘legitimate peripheral non‐participation’ is a prerequisite for students to observe and gather enough information and courage to involve themselves in clinical work (Lave & Wenger 1991, Spouse 1998a). Gradually students involved themselves in ‘legitimate peripheral participation’ as they managed to latch on to a local ward nurse on shift to take part in minor tasks or simple procedures at hand. However, due to the large number of nurses in the large wards it was often frustrating for the students to have to start all over with ‘legitimate peripheral non‐participation’ the next day and introduce themselves again as they met with new unfamiliar nurses. During tea and lunch breaks, students seldom socialized with the local nurses.

As noted above, a significant turning point for students' inclusion in the Bangladeshi nursing CoP took place when they changed their placement to the smaller community hospital in Matlab where the students lived in a guesthouse at the hospital premises, socialized and had lunch together with members of the small team of local nurses. They easily got an overview of the small rural hospital and on arrival they were introduced and welcomed by the local nursing CoP. The sponsorship from the local nurses in the Matlab wards allowed them to gain courage and enabled them to feel safe enough to become regular legitimate peripheral participants. This experience strengthened their confidence and helped them become more involved in the clinic on their return back to Dhaka hospital. For instance, students supported each other in pairs and performed cannulation, administered intra‐venous fluid, assessed grades of dehydration and supported relatives at the triage.

At times students opted not to participate in clinical practice. This ‘voluntary legitimate non‐participation’ happened when the students faced a clinical practice, which they perceived as malpractice, or an ethical dilemma, as a result they dissented. Most of the time students did so in silence. However, sometimes they politely confronted local nurses with their thoughts of what they perceived as breach in procedures. Such discussion usually were revealing for the students. The students always used the proto‐CoP for de‐briefing and reflection after a dissent.

### Limitations

Additional data are needed and follow‐up interviews would provide more depth to the understanding of how nursing students reintegrate in Norway. An exploration of how nursing students cope with reintegration coming home and how they managed to incorporate their insights from a clinical placement abroad in their professional nursing practice in Norway needs to be investigated further in a future study.

## Concluding remarks

Above, we have illustrated how marginality and ‘peripheral participation’ trigger insight and reflection. The students' role in clinical placement was balanced between being an observer and being a participant. The challenging but advantageous position of the peripheral students was heightened further due to the lack of one‐to‐one supervision in the clinic and due to socio‐cultural differences encountered in Bangladesh. Their previous experience with PBL and group learning was an asset which made them more resilient and helped to cope during their clinical placement in Bangladesh (Ehrenberg & Häggblom 2007, Chen 2011). Clinical placement in groups has been launched in Norway and elsewhere as an alternative approach (Ranse & Grealish 2007, Medby & Haugan 2012).

As the students returned, back to Norway, they experienced marginality and a reversed culture ‘shock’, a break in their learning trajectory, a brake with the proto‐CoP and some difficulties with integration in the Norwegian nursing CoP. Some measures that can help students to incorporate their insights from a clinical placement abroad in their professional nursing practice as they join their Norwegian CoP have to be developed. During the regular audit response of their clinical placement, abroad students expressed their satisfaction. They recommended that the three focus group interviews should be continued for all students who do clinical placement abroad as standard offer, a valuable possibility to pre‐brief, peri‐brief and a de‐brief.

## Conflicts of interest

None declared.

## Author contributions

All authors have agreed on the final version and meet at least one of the following criteria [recommended by the ICMJE (http://www.icmje.org/recommendations/)]:

- substantial contributions to conception and design, acquisition of data, or analysis and interpretation of data;
- drafting the article or revising it critically for important intellectual content.
